# Synthesis of Porous Lithium Ion Sieve with High Purity for Li^+^ Adsorption

**DOI:** 10.3390/ma18102373

**Published:** 2025-05-20

**Authors:** Jing Zhu, Xiyun Yang, Yongqiang Huang, Rongzheng Yao

**Affiliations:** School of Metallurgy and Environment, Central South University, Changsha 410083, China

**Keywords:** lithium ion sieve, solid-phase synthesis, adsorption capacity

## Abstract

With the depletion of solid lithium ore, extracting lithium from salt lake brine has become a critical focus for future endeavors. A four-step method was used to synthesize high-purity H_1.6_Mn_1.6_O_4_ for extracting Li^+^. Porous cubic Mn_2_O_3_ was hydrothermally synthesized with carbon spheres and surfactants as templates. Then, it was converted to LiMnO_2_ by calcining with Li_2_CO_3_. After roasting and acid pickling, H_1.6_Mn_1.6_O_4_ was successfully synthesized. The impacts of calcination temperature, Li/Mn molar ratio and glucose addition on LiMnO_2_ composition, loss percentage of dissolved Mn in precursor, and the adsorption characteristics of the lithium ion sieve were studied. Glucose inhibited the formation of LiMn_2_O_4_ and promoted the formation of pure LiMnO_2_. The resulting precursor without impurities showed porous structure. After acid pickling, H_1.6_Mn_1.6_O_4_ showed a high-adsorption performance and excellent cycle performance. After five cycles, adsorption capacity remained above 30 mg/g, and the loss percentage of dissolved Mn stabilized at about 1%. The Li^+^–H^+^ exchange conformed to pseudo-second-order adsorption dynamics and the Langmuir adsorption isotherm equation, indicating that the adsorption process can be classified as monolayer chemical adsorption.

## 1. Introduction

As lithium battery and new energy industries experience rapid growth [1,2,3,4], the demand for lithium in China and the world has soared [5,6,7,8]. The lithium resource in China is generally categorized into solid lithium ore and liquid brine [9,10,11]. Notably, approximately 80% of the total lithium reserve is found in salt lakes [12,13,14]. Due to the exhaustion of solid lithium ore, the extraction and recovery of lithium from salt lake brine has attracted significant attention. At present, several methods are used to separate and extract lithium, including membrane separation [15,16], solvent extraction [17,18,19], and adsorption methods [20,21,22]. Among these, the adsorption method stands out as an ideal and prospective approach due to its excellent separation effect, environmental sustainability, and economic advantages [14]. Compared with other adsorbents, the Mn-based adsorbent has high selectivity for lithium and superior adsorption properties. It typically includes various kinds of lithium ion sieves, such as HMn_2_O_4_ [23], H_4_Mn_5_O_12_ [24], and H_1.6_Mn_1.6_O_4_ [25,26]. H_1.6_Mn_1.6_O_4_ is synthesized by roasting LiMnO_2_ to obtain the precursor Li_1.6_Mn_1.6_O_4_ and then replacing Li^+^ with H^+^, resulting in the ion exchange to adsorb Li^+^ and exhibiting the maximum lithium adsorption capacity. Chitrakar [27] developed and utilized a H_1.6_Mn_1.6_O_4_ adsorbent for recovering lithium from seawater in the initial stages. Hayashi [28] synthesized a H_1.6_Mn_1.6_O_4_ rod with an adsorption capacity of 5.6 mmol g^−1^. And researchers have also explored the Li^+^ extraction mechanism [29,30,31]. However, the composition of LiMnO_2_ and the morphology of Li_1.6_Mn_1.6_O_4_ have great effect on the performance of H_1.6_Mn_1.6_O_4_ [25]. The presence of a small amount of Mn^3+^ in the precursor could lead to an unstable spinel structure and deterioration in cycling performance due to disproportionation during acid pickling [32].

Adsorbents characterized by numerous different pores structures promote effective adsorption because they expose additional adsorption sites for ion exchange [33]. And the distribution of appropriate pore sizes can effectively reduce the diffusion pathway for Li^+^, thereby enhancing the overall adsorption performance. Simultaneously, the Mn-based lithium ion sieve with porous structure has a relatively large specific surface area, which is conducive to facilitating Li^+^ migration and improving the adsorption rate [34,35]. Xu [36] synthesized a lithium ion sieve using attapulgite with a large surface area, which provided an adsorption capacity of 29.18 mg/g. Finally, porous structures feature a large gas channel, which is beneficial to oxygen diffusion and provides thorough contact with the bulk of crystal LiMnO_2_. As a result, LiMnO_2_ can be completely oxidized to obtain high-purity Li_1.6_Mn_1.6_O_4_ with traces of Mn^3+^ [23,25].

In this study, porous Mn_2_O_3_ was firstly synthesized by template method. Then, Mn_2_O_3_, Li_2_CO_3_, and glucose were calcined by carbothermal reduction-assisted solid-phase method [37] to obtain pure LiMnO_2_. Finally, LiMnO_2_ was converted to the precursor through roasting, and then, the lithium ion sieve was synthesized after acid pickling.

## 2. Materials and Methods

### 2.1. Materials

The reagents C_6_H_12_O_6_, Li_2_CO_3_, HCl, and C_2_H_5_OH were from Sinopharm Group Chemical Reagent Co., Ltd. (Shanghai, China). The reagents Mn(CH_3_COO)_2_, polyvinylpyrrolidone (PVP), and CO(NH_2_)_2_ were supplied by Aladdin Industrial Co., Ltd. (Shanghai, China). These were all AR reagents. The salt lake brine was from Lop Nor Lake in the Xinjiang Uygur Autonomous Region of China.

### 2.2. Methods

#### 2.2.1. Synthesis of Porous Cubic Mn_2_O_3_

Firstly, carbon sphere [38] was prepared by hydrothermal treatment of 1 mol/L glucose solution at 200 °C for 24 h. The porous Mn_2_O_3_ was synthesized according to the method of Shao Y [39]. Urea CO(NH_2_)_2_ as precipitant and Mn(CH_3_COO)_2_ as manganese salt were used to hydrothermally synthesize MnCO_3_/C composites with carbon spheres and surfactant PVP (PVP is a nonionic polymer compound) as templates. Next, 2 g carbon spheres and 10 g PVP were dispersed into water and stirred for 12 h. Then, CO(NH_2_)_2_ and Mn(CH_3_COO)_2_ were added according to a molar ratio of manganese salt to precipitant of 1:40. After vigorous stirring, the suspension was transferred to an autoclave and hydrothermally reacted at 90 °C for 12 h. MnCO_3_/C composites were obtained after filtering, washing, and drying. Finally, MnCO_3_/C was transformed into porous Mn_2_O_3_ by calcining at 600 °C for 6 h in a muffle furnace [34].

#### 2.2.2. Synthesis of LiMnO_2_ and Precursor Li_1.6_Mn_1.6_O_4_

Li_2_CO_3_, porous Mn_2_O_3_, 5% (equivalent to the mass of Mn_2_O_3_) of glucose, and an appropriate amount of anhydrous ethanol were thoroughly ground to fully and evenly mix them. Then, the mixture was calcined in a closed tube furnace with nitrogen blowing at 750 °C for 6 h to obtain LiMnO_2_. Finally, LiMnO_2_ was roasted at 450 °C for 6 h to synthesize the precursor Li_1.6_Mn_1.6_O_4_ [40].

#### 2.2.3. Synthesis of Lithium Ion Sieve

A small quantity of Li_1.6_Mn_1.6_O_4_ was added to 0.5 mol/L hydrochloric acid solution. The Mn-based lithium ion sieve was successfully synthesized after acid pickling at 30 °C for 4 h.

#### 2.2.4. Adsorption and Cycling Performance

The lithium ion sieve was put into a certain volume of brine and stirred to facilitate Li^+^ adsorption. Adsorption capacity (Q) was calculated by following Equation (1):

(1) $$Q=\frac{V(C_{0}-C_{1})}{m},$$

where Q represents the Li^+^ adsorption capacity of H_1.6_Mn_1.6_O_4_ (mg·g^−1^); C_0_ and C_1_ represent the concentration of Li^+^ in solution before adsorption and after adsorption, respectively (mg·L^−1^); V represents the volume of salt lake brine (L); m represents the mass of H_1.6_Mn_1.6_O_4_ (g).

The loss percentage of dissolved Mn (R_Mn_) in acid pickling was calculated by Equation (2):

(2) $$R_{\mathrm{Mn}}=\frac{C_{\mathrm{Mn}}V}{m_{s}W},$$

where R_Mn_ represents the loss percentage of dissolved Mn (%); C_Mn_ represents the concentration of Mn^2+^ in filtrate after acid pickling (mg·L^−1^); V represents the volume of hydrochloric acid (L); m_s_ represents the mass of the precursor (g); W represents the mass fraction of Mn in the precursor (%).

The acid pickling and adsorption of Li^+^ were repeated for several times, and then, the cycling performance was determined. In each acid pickling process, the precursor Li_1.6_Mn_1.6_O_4_ was put into 0.5 mol/L hydrochloric acid solution for delithiation. After filtering, washing, and drying, H_1.6_Mn_1.6_O_4_ was transferred into salt lake brine at 30 °C for 24 h to adsorb Li^+^. These above operations were considered as one complete cycle. After this process, the concentrations of Mn and Li were measured to calculate the loss percentage of dissolved Mn and adsorption capacity in every cycle using Equation (1) and Equation (2), respectively.

#### 2.2.5. Evaluation of Adsorption Selectivity

The adsorption selectivity of H_1.6_Mn_1.6_O_4_ is a crucial indicator under competitive conditions, particularly in the presence of co-existing ions such as Na^+^ and Mg^2+^. A small amount of H_1.6_Mn_1.6_O_4_ was put into brine for adsorption. Subsequently, the concentrations of mental ions were measured to calculate the adsorption capacity and distribution coefficient (K_d_) as follows:

(3) $$K_{d}=\frac{(C_{0}-C_{1})V}{mC_{1}},$$

where K_d_ represents the distribution coefficient of different ions (mL·g^−1^); C_0_ and C_1_ represent the concentration of ions in solution before adsorption and after adsorption, respectively (mg·L^−1^); V represents the volume of salt lake brine (L); m represents the mass of H_1.6_Mn_1.6_O_4_ (g);

#### 2.2.6. Evaluation of Adsorption Kinetics

A small amount of H_1.6_Mn_1.6_O_4_ was put into brine at room temperature for certain time and sampled at regular interval to measure the concentration of Li^+^ for calculating adsorption capacity. The H^+^–Li^+^ exchange process was simulated using pseudo-first-order and pseudo-second-order dynamics models and the intraparticle diffusion model, respectively.

The equations of three adsorption kinetics are as follows:

(4) $$\mathrm{lg}(Q_{e}-Q_{t})=\mathrm{lg}Q_{e}-\frac{t\cdot k_{1}}{2.303},$$

(5) $$\frac{t}{Q_{t}}=\frac{1}{k_{2}\cdot {Q_{e}}^{2}}+\frac{1}{Q_{e}}t,$$

(6) $$\mathrm{and}\;Q_{t}=K_{i}\cdot t^{0.5}+C$$

where Q_t_ represents the adsorption capacity of H_1.6_Mn_1.6_O_4_ at given time (mg·g^−1^); Q_e_ represents the adsorption capacity of H_1.6_Mn_1.6_O_4_ at equilibrium (mg·g^−1^); t represents adsorption time (h); k_1_, k_2_, and K_i_ represent the adsorption kinetic rates, respectively; C is the intercept.

#### 2.2.7. Determination of Adsorption Isotherm

A small amount of H_1.6_Mn_1.6_O_4_ was put into LiCl solutions with different concentrations at room temperature for 24 h to adsorb Li^+^. The adsorption isotherm was simulated using Langmuir and Freundlich models, respectively.

The equations of the two models are as follows:

(7) $$\frac{C_{e}}{Q_{e}}=\frac{1}{K_{L}Q_{m}}+\frac{C_{e}}{Q_{m}}$$

(8) $$\mathrm{and}\;\mathrm{lg}Q_{e}=\frac{1}{n}\mathrm{lg}C_{e}+\mathrm{lg}K_{F},$$

where C_e_ represents the Li^+^ equilibrium concentration (mg·L^−1^); Q_e_ represents the adsorption capacity of H_1.6_Mn_1.6_O_4_ at equilibrium (mg·g^−1^); Q_m_ represents the theoretical maximum adsorption capacity of H_1.6_Mn_1.6_O_4_ (mg·g^−1^); K_L_ and K_F_ represent the adsorption isothermal constants, respectively.

#### 2.2.8. Analysis and Characterization

The concentrations of ions were quantified after acid pickling and adsorption using inductively coupled plasma spectrometer (ICP, Thermo Fisher Scientific, Waltham, MA, USA). Phase composition was analyzed using X-ray diffractometer (XRD, PANalytical B.V., Almelo, The Netherlands). The morphology of the precursor was characterized using scanning electron microscope (SEM, JEOL, Tokyo, Japan).

## 3. Results

### 3.1. Properties of Porous Mn_2_O_3_

**Figure 1:**
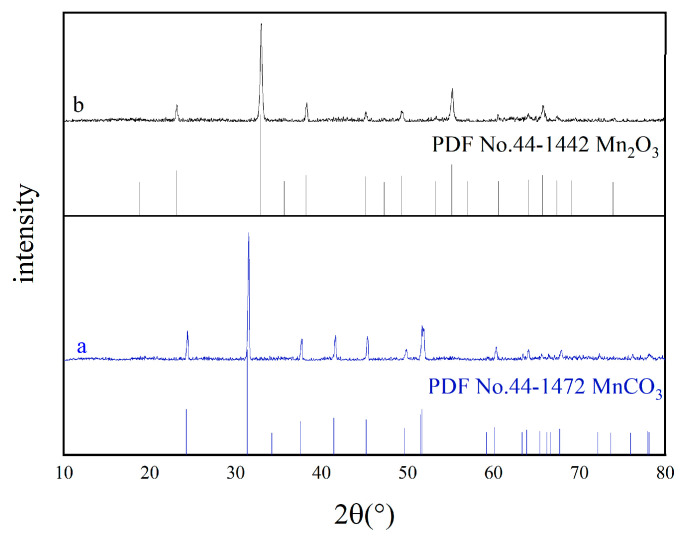
XRD patterns of (**a**) MnCO_3_/C and (**b**) Mn_2_O_3_.

XRD patterns of MnCO_3_/C and Mn_2_O_3_ are presented in Figure 1. Its diffraction peak is very sharp in Figure 1a, which is consistent with MnCO_3_(PDF NO. 44-1472). There are no carbon and other impurity diffraction peaks. This indicates the synthesized MnCO_3_ has good crystallinity and purity. The calcined Mn_2_O_3_ also shows sharp peaks identified with cubic α-Mn_2_O_3_(PDF NO. 24-0508), meaning that it is highly crystalline and pure.

**Figure 2:**
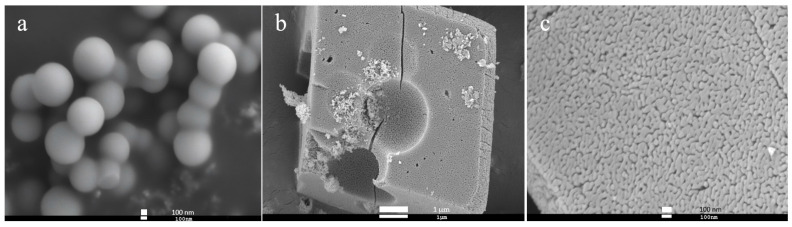
SEM images of (**a**) carbon spheres, (**b**) cubic Mn_2_O_3_, (**c**) and the partial enlargement.

Figure 2 illustrates the morphologies of carbon spheres and cubic Mn_2_O_3_. According to the SEM image presented in Figure 2a, it can be observed that carbon spheres are intact and have uniform size ranging from 800 nm to 1.5 μm. From Figure 2b, Mn_2_O_3_ maintains a complete cubic shape with size around 6–8 μm. However, there are numerous spherical concaves on the particle surface, and the dimensions of these concaves are similar to those of carbon spheres. It can be inferred that these concaves were formed during the calcining process of carbon spheres. Additionally, as shown in the partially enlarged image (Figure 2c), the Mn_2_O_3_ cubic structure has some interconnected channels with a width of more than 20 nm. These channels may result from both the decomposition of MnCO_3_ and the oxidation of carbon spheres during calcination [41]. The release of CO_2_ contributes to the channel formation. Furthermore, PVP as a soft template also releases CO_2_ during this process [42], leading to the formation of more gaps on the surface of the Mn_2_O_3_ cubic structure.

### 3.2. Synthesis of LiMnO_2_

#### 3.2.1. Optimization of Calcination Temperature

**Figure 3:**
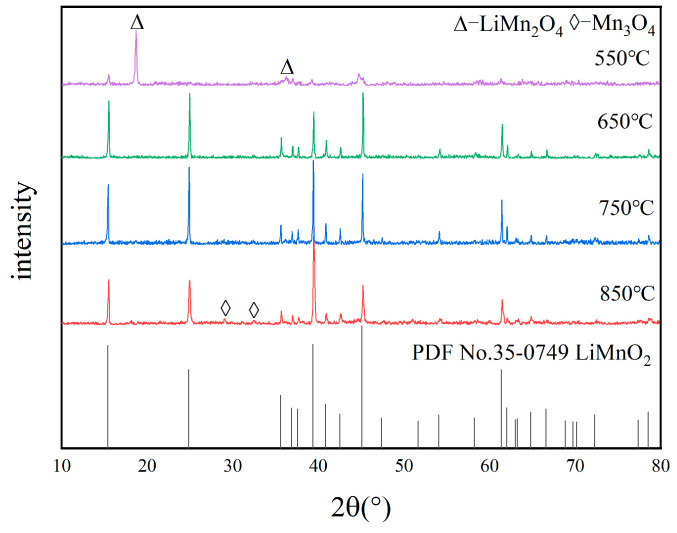
XRD patterns of samples at different calcination temperatures.

Figure 3 presents XRD patterns of samples obtained at various calcination temperatures. As shown, a pure phase of LiMnO_2_ was obtained at 650 °C and 750 °C. However, only a small amount of LiMnO_2_ was produced at 550 °C, with the majority being LiMn_2_O_4_. The diffraction peaks of LiMnO_2_ are weak, and crystallization is incomplete at 550 °C. The intensity and purity of diffraction peaks also increases when the calcination temperature rises, facilitating the formation of a pure product. At a temperature of 850 °C, a small amount of Mn_3_O_4_ impurity appears, which results from the decomposition of Mn_2_O_3_.

#### 3.2.2. Optimization of Molar Ratio of Lithium to Manganese

**Figure 4:**
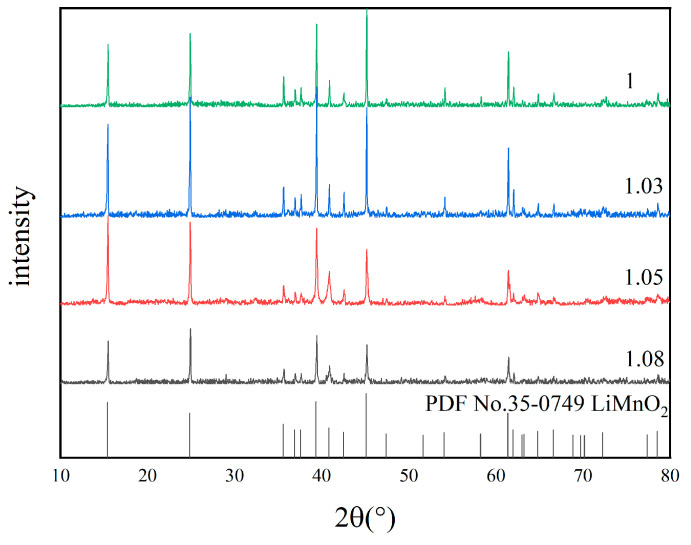
XRD patterns of samples with different Li/Mn ratios.

Figure 4 illustrates the XRD patterns of samples synthesized at various molar ratios of lithium to manganese (Li/Mn). It can be observed from Figure 4 that pure LiMnO_2_ can be successfully obtained no matter what the Li/Mn ratio is. But as the Li/Mn ratio reaches 1.03, the resulting diffraction peaks are sharper compared to those with other ratios, and the crystallization is also more complete. When the Li/Mn ratio is 1.05 and 1.08, the intensity of the LiMnO_2_ diffraction peak is weaker compared to that observed at a Li/Mn ratio of 1.03. An excessive Li/Mn ratio results in a large amount of Li^+^ entering into the structure, even affecting the transition element layer [32]. This impacts the structural integrity of LiMnO_2_, resulting in poor crystallinity and a mixing phenomenon of cations within the precursor [43]. From an economic perspective, a Li/Mn ratio of 1.03 represents an optimal choice.

#### 3.2.3. Optimization of Glucose Content

**Figure 5:**
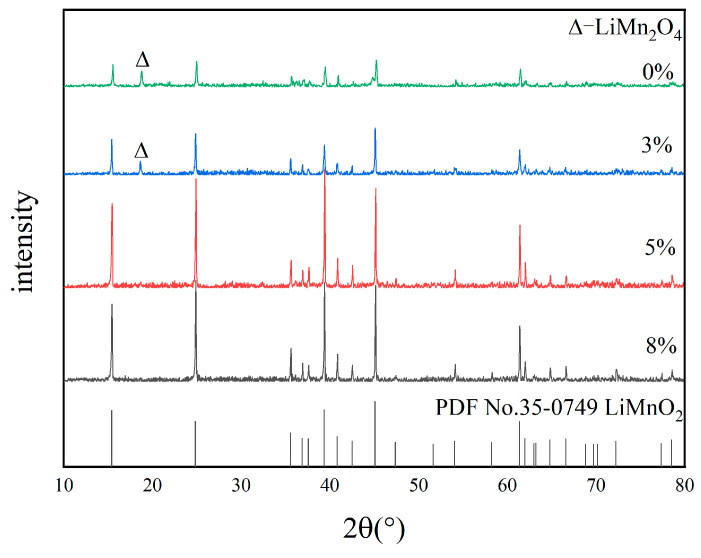
XRD patterns of samples with different glucose amounts.

Figure 5 presents the XRD patterns of LiMnO_2_ synthesized with various amount of glucose (relative to Mn). As shown in Figure 5, the intensity of impurity peaks is weaker, and the LiMnO_2_ main peak becomes sharper with the increase in glucose content. LiMn_2_O_4_ impurity peaks are observed for the blank and 3% glucose. In contrast, pure LiMnO_2_ can be obtained when the glucose content exceeds 5%. Glucose does not affect the composition of subsequent products. The presence of LiMn_2_O_4_ impurity primarily arises from the decomposition of a portion of Mn_2_O_3_ during the high-temperature synthesis process of LiMnO_2_, leading to small quantities of Mn_3_O_4_ and O_2_ [37]. This part of Mn_3_O_4_ and O_2_ subsequently reacts with lithium to form LiMn_2_O_4_. Simultaneity, excess O_2_ when glucose is insufficient may also oxidize a minor amount of LiMnO_2_ into LiMn_2_O_4_. The relevant chemical equations are shown in (9) and (10):

(9) $$6{\mathrm{Mn}}_{2}O_{3}=4{\mathrm{Mn}}_{3}O_{4}+O_{2}↑$$

(10) $$\mathrm{and}\;8{\mathrm{Mn}}_{3}O_{4}\;+\;5O_{2}\;=\;6{\mathrm{Li}}_{2}{\mathrm{CO}}_{3}\;=\;12{\mathrm{LiMn}}_{2}O_{4}+6{\mathrm{CO}}_{2}$$

In the high-temperature solid-phase reaction process, the decomposition of Mn_2_O_3_ is unavoidable. Therefore, altering process parameters, such as the calcination temperature or the molar ratio of Li/Mn, does not completely eliminate the impurity of LiMn_2_O_4_. Impurities in LiMnO_2_ can lead to impure composition during the synthesis of the precursor Li_1.6_Mn_1.6_O_4_. This adversely affects the structure and purity of the lithium ion sieve, resulting in a decreased adsorption capacity and an increased Mn dissolution loss percentage. Although HMn_2_O_4_ has a certain adsorption capacity, its adsorption properties are inferior to those of H_1.6_Mn_1.6_O_4_. Therefore, the presence of impurities in LiMnO_2_ may result in an unsatisfactory adsorption performance of the subsequently formed lithium ion sieve. Yu [44] also confirmed the view by analyzing the obvious positive correlation between the adsorption density of Li and the content of H_4_Mn_5_O_12_. So, the addition of glucose is essential. Firstly, glucose can directly consume O_2_ within the reaction environment, thereby protecting LiMnO_2_ from oxidation. Secondly, glucose can reduce partial impurities back into LiMnO_2_. Thus, two functions synergistically contribute to synthesizing pure LiMnO_2_. Glucose acts as a reducing agent to protect LiMnO_2_ from oxidation and does not remain within the adsorbent after synthesis. Consequently, high-purity LiMnO_2_ can be synthesized through carbothermal reduction-assisted solid-phase reactions.

### 3.3. Characterization of Li_1.6_Mn_1.6_O_4_

**Figure 6:**
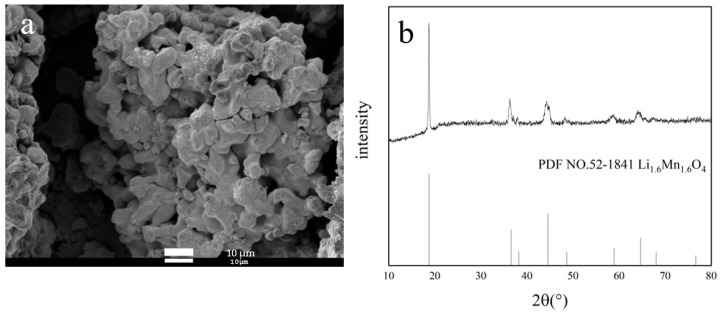
(**a**) SEM image and (**b**) XRD pattern of Li_1.6_Mn_1.6_O_4_.

According to the above results, the optimal synthetic conditions are as follows: calcination temperature of 750 °C, molar ratio of Li/Mn of 1.03, and addition of glucose at 5%. Li_1.6_Mn_1.6_O_4_ can be obtained by roasting LiMnO_2_ at 450 °C for 6 h. Figure 6 illustrates the SEM image and XRD pattern of Li_1.6_Mn_1.6_O_4_. As shown in Figure 6a, the particles of Li_1.6_Mn_1.6_O_4_ aggregate into cubic structures with obvious gaps. Some gaps conjunct together and even form holes. The features may facilitate the formation of suitable channels that allow for the free movement of Li^+^ in and out without destroying the spinel structure [45]. The structural framework closely resembles cubic Mn_2_O_3_ (Figure 2b). Furthermore, as presented in Figure 6b, the diffraction peak is consistent with Li_1.6_Mn_1.6_O_4_(PDF NO.52-1841), indicating that the product is composed of Li_1.6_Mn_1.6_O_4_ monophase with cubic crystal structure.

## 4. Discussion

### 4.1. Adsorption Performance of H_1.6_Mn_1.6_O_4_

**Figure 7:**
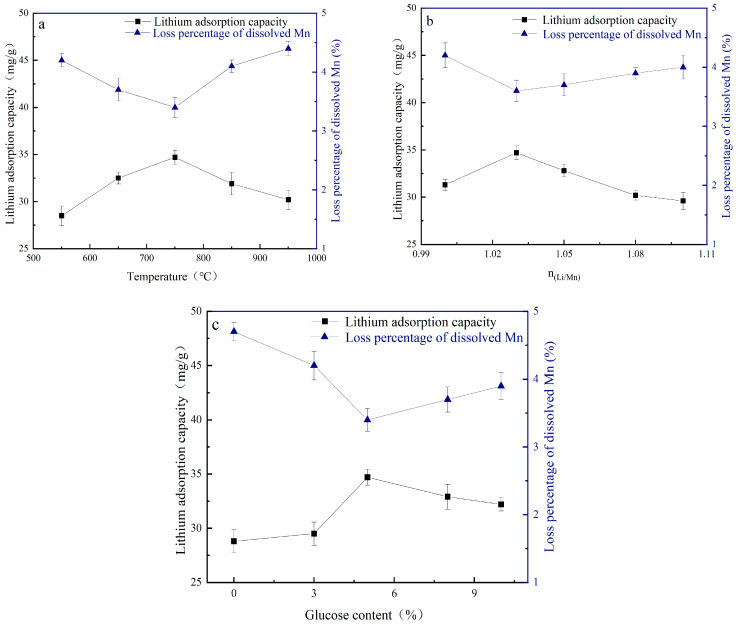
The loss percentage of dissolved Mn and corresponding adsorption capacity at different (**a**) calcination temperatures, (**b**) molar ratios of Li/Mn, and (**c**) glucose contents.

Figure 7 shows the loss percentage of dissolved Mn of the precursor by roasting LiMnO_2_ and the adsorption capacity for Li^+^ at various calcination temperatures, molar ratios of Li/Mn, and glucose contents. In Figure 7a, H_1.6_Mn_1.6_O_4_ shows a maximum adsorption performance of about 34.7 mg/g and a minimum Mn dissolution loss of about 3.4% at 750 °C. The change of the Mn dissolution loss percentage with calcination temperature is due to the fact that a low calcination temperature leads to LiMn_2_O_4_ formation and a high temperature to Mn_3_O_4_, as shown in Figure 3. Those two species contain Mn^3+^ and cause a disproportion reaction in hydrochloric acid, resulting in the formation of Mn^4+^ and Mn^2+^. The release of Mn^2+^ into solution leads to a relatively high percentage of Mn dissolution loss. Figure 7b shows the Li/Mn ratio has a small effect on the adsorption performance and loss percentage of dissolved Mn, and this response is consistent with the result in Figure 4. However, the adsorption performance and loss percentage of dissolved Mn have great dependence on the glucose content. When it is less than 5%, a high glucose content is beneficial for improving the adsorption capacity and structure stability, as shown in Figure 7c. Further increasing the glucose content has no obvious effect compared with 5% glucose. At same time, it can be observed that the lithium ion sieve obtained by roasting LiMnO_2_ without impurities and acid pickling has a higher adsorption capacity and lower loss percentage of dissolved Mn than that obtained by roasting LiMnO_2_ containing impurities and acid pickling. These results indicate that the composition of LiMnO_2_ has a great difference from the performance of the Mn-based lithium ion sieve [25].

Table 1 describes the performance of other solid-phase synthetic non-porous lithium ion sieves. Compared to the references, the Li^+^ adsorption capacity is improved. This enhancement can be attributed to the porous cubic structure of H_1.6_Mn_1.6_O_4_ particles. A porous structure is conducive to Li^+^ migration and exchange, leading to a higher adsorption capacity [46]. Li [47] also confirmed the view by synthesizing 3D macroporous–mesoporous H_4_Ti_5_O_12_ and porous H_4_Ti_5_O_12_. They found higher Li^+^ adsorption capacity compared with nonporous H_4_Ti_5_O_12_ because of the highly interconnected porous channel.

### 4.2. Cycling Performance

**Figure 8:**
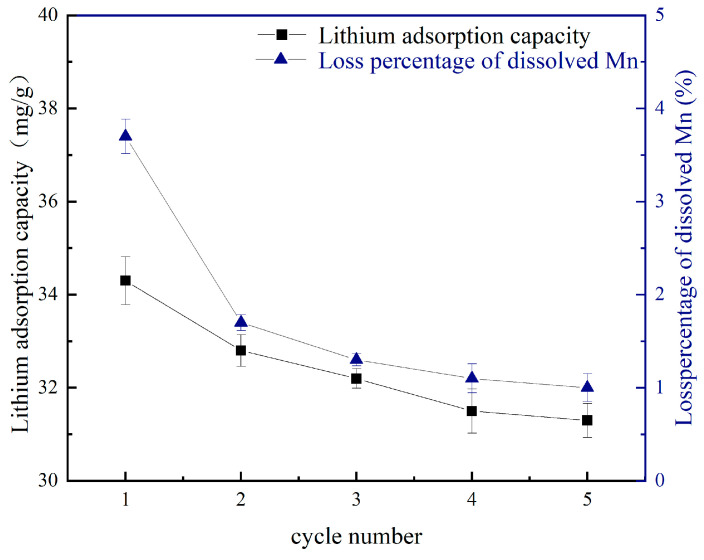
Loss percentage of dissolved Mn and corresponding adsorption capacity of precursors during circulation.

The loss percentage of dissolved Mn during acid pickling and Li^+^ adsorption capacity were determined over multiple cycles. The results are illustrated in Figure 8. During the initial three cycles, the loss of dissolved Mn is due to the Mn^3+^ disproportion reaction in hydrochloric acid [51]. After three cycles, nearly all Mn^3+^ was dissolved, and the percentage of Mn dissolution loss presented a constant value approximately 1%. This stability can be attributed to the formation of high-purity precursor Li_1.6_Mn_1.6_O_4_ following the roasting of LiMnO_2_ without impurities. Figure 8 also demonstrates that the lithium adsorption capacity decreases as the number of cycles increases. This reduction can be attributed to the H^+^–Li^+^ ion exchange process during the acid pickling, where H^+^ possesses smaller ionic radius compared to Li^+^ [52]. During the adsorption process, this exchange between Li^+^ and H^+^ inevitably leads to a contraction of the crystal structure. Consequently, this phenomenon hinders both diffusion and embedding of Li^+^ into the crystal lattice, ultimately leading to a decreased lithium adsorption capacity. After five cycles, the adsorption capacity remained more than 30 mg/g, and recovery rate was about 85%. The resulting lithium ion sieve therefore exhibits high stability during cycling.

### 4.3. Adsorption Selectivity

**Figure 9:**
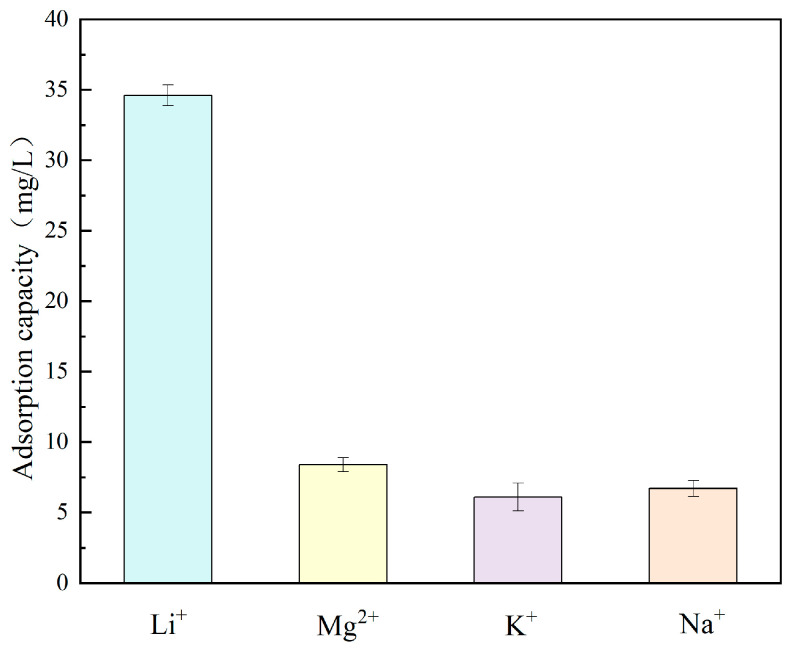
Adsorption capacity for various ions.

The existence of various ions in salt lake brine has a significant impact on the Li^+^ adsorption effect of the lithium ion sieve. Figure 9 demonstrates the adsorption capacity of H_1.6_Mn_1.6_O_4_ for Li^+^, Mg^2+^, K^+^, and Na^+^. H_1.6_Mn_1.6_O_4_ has the highest adsorption capacity for Li^+^ compared to Mg^2+^, K^+^, and Na^+^, and the distribution coefficient of Li^+^ is much larger than that of other ions. These results indicate the synthesized lithium ion sieve exhibits exceptional selectivity for Li^+^.

### 4.4. Adsorption Kinetics

**Figure 10:**
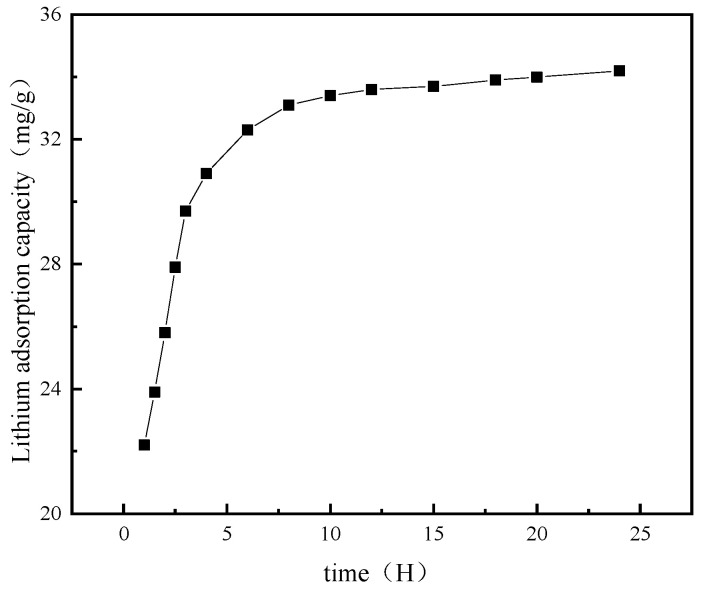
The variation in adsorption capacity over time.

The variation in adsorption capacity over time is illustrated in Figure 10. As shown, adsorption capacity progressively enhances over time. The initial adsorption rate is notably rapid during the first stage but subsequently slows down. This is due to the fact that there are numerous lithium vacancies exhibiting a memory effect within the spinel structure after acid pickling. Additionally, a significant concentration gradient of Li^+^ between brine and the interior of ion sieve is beneficial for Li^+^ to enter into the crystal lattice and cause exchange with H^+^ [53]. However, H^+^ in H_1.6_Mn_1.6_O_4_ enters into the solution as time goes by, leading to an increase in the concentration of H^+^. Simultaneously, as Li^+^ is adsorbed into the ion sieve, there is a decline in the Li^+^ concentration in brine. These factors hinder both the Li^+^–H^+^ exchange and overall adsorption processes. Thus, the initial rate of adsorption is fast, while the subsequent adsorption rate gradually becomes gentle.

**Figure 11:**
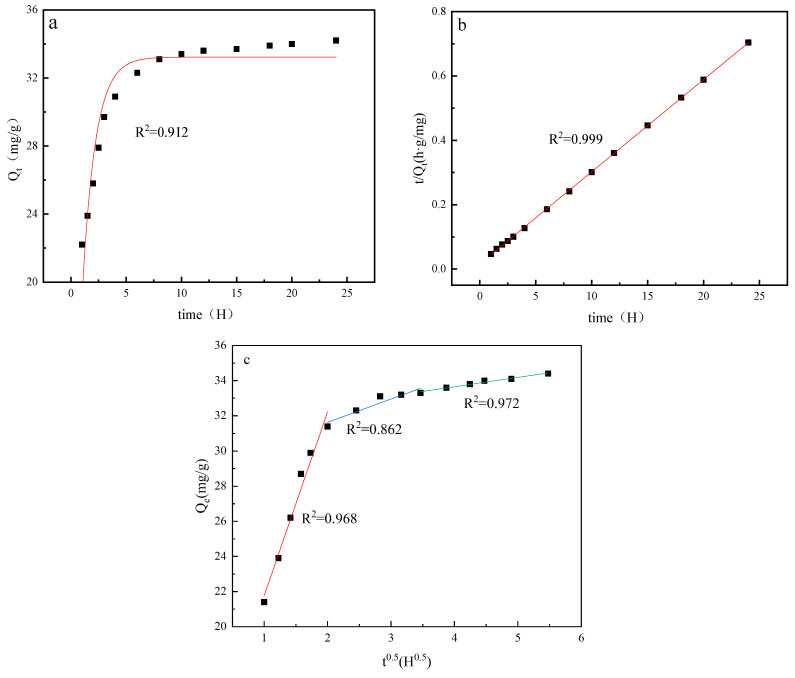
Fitting results of (**a**) pseudo-first-order dynamics, (**b**) pseudo-second-order dynamics, and (**c**) intraparticle diffusion model.

Figure 11 illustrates three kinetic models for adsorption process of lithium ion sieve. Using the pseudo-first-order adsorption dynamics model gives a linear correlation coefficient of about only 0.912. However, the linear correlation coefficient increases significantly to 0.999, and the adsorption constant K is 0.051 based on the pseudo-second-order adsorption dynamics model. Figure 11c shows that the resulting straight lines do not pass through the origin, and the correlation coefficients are lower that of the pseudo-second-order kinetic model. These results show the process of ion exchange between Li^+^ and H^+^ is not consistent with the pseudo-first-order dynamics model and intraparticle diffusion model but closely conforms to the pseudo-second-order adsorption dynamics model. These findings suggest the adsorption process of the lithium ion sieve is primarily controlled by chemical adsorption.

### 4.5. Adsorption Isotherm

**Figure 12:**
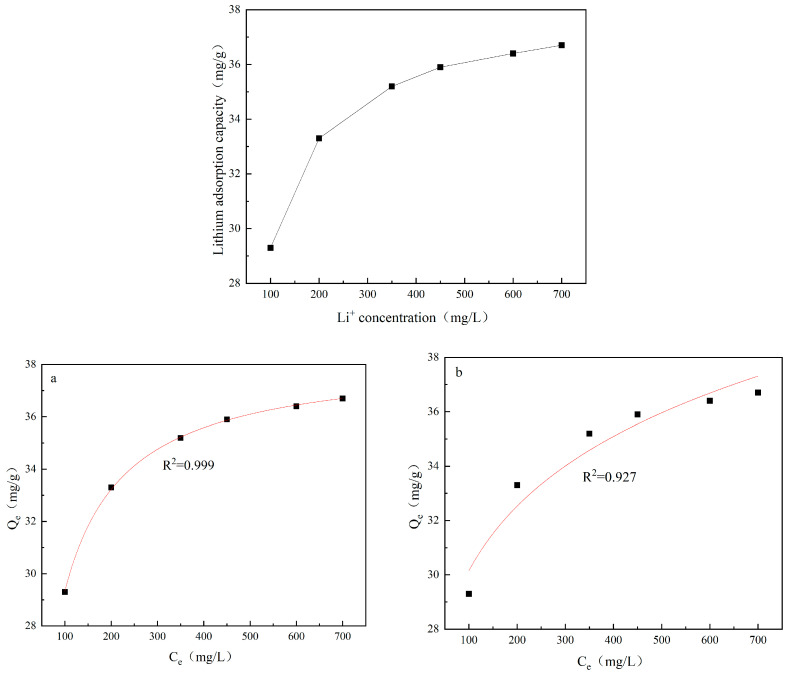
Adsorption isotherm and fitting results of the (**a**) Langmuir and (**b**) Freundlich equations.

Figure 12 presents the adsorption isotherm of the lithium ion sieve and the fitting results with Langmuir and Freundlich models. With the increase in Li^+^ concentration in the solution, the adsorption capacity of H_1.6_Mn_1.6_O_4_ also correspondingly increases. The correlation coefficient based on the Langmuir equation is 0.999, while that of the Freundlich model is just 0.927. The results indicate that the adsorption process of H_1.6_Mn_1.6_O_4_ belongs to monolayer adsorption.

## 5. Conclusions

Porous Mn_2_O_3_ was hydrothermally synthesized and calcined with Li_2_CO_3_, using a carbothermal reduction-assisted solid-phase method to obtain LiMnO_2_. Subsequently, a lithium ion sieve was synthesized after roasting LiMnO_2_ and acid pickling. The optimum synthesis conditions of LiMnO_2_ are as follows: calcination temperature of 750 °C, Li/Mn of 1.03, and addition of 5% glucose. The carbothermal reduction method protects manganese from oxidation and yields high-purity LiMnO_2_. The resulting Mn-based lithium ion sieve has an adsorption capacity of 35 mg/g. After five cycles, the dissolution percentage of Mn is stable at approximately 1%. Simultaneously, the adsorption capacity remains above 30 mg/g, demonstrating excellent cycling performance and structural stability.

## Figures and Tables

**Table 1:** Performance of other solid-phase synthetic non-porous lithium ion sieves.

Precursor	Adsorption Capacity (mg/g)	Loss Percentage of Dissolved Mn (%)	Cite
LiMn_2_O_4_	30.9	25	[48]
LiMn_2_O_4_	32.8	-	[49]
Li_1.6_Mn_1.6_O_4_	29.4	1.62	[50]
Li_1.6_Mn_1.6_O_4_	34.7	3.4	This article

## Data Availability

The original contributions presented in this study are included in the article. Further inquiries can be directed to the corresponding author.
